# A case of convexity non-aneurysmal subarachnoid hemorrhage caused by cerebral sinus thrombosis

**DOI:** 10.1186/s12245-024-00712-3

**Published:** 2024-10-10

**Authors:** Ali Abasi, Asra Moradkhani, Shiva Rahimi, Hannah Magrouni

**Affiliations:** 1 Student of the Research Committee, Kurdistan University of Medical Sciences, Sanandaj, Iran; 2 Department of Neurology, Kurdistan University of Medical Sciences, Sanandaj, Iran

**Keywords:** Subarachnoid hemorrhage, Cerebral sinus thrombosis, Case report, Convexity SAH

## Abstract

**Background:**

Convexity subarachnoid hemorrhage (cSAH) is an uncommon presentation of subarachnoid bleeding, referring to bleeding more localized to the convexities of the brain. The diagnosis of cerebral venous sinus thrombosis (CVST) can be difficult especially when patients initially present with cSAH. The authors present a case and then discuss the pathophysiology and management.

**Case presentation:**

A 56-year-old woman with a previous history of hypertension and ischemic heart disease presented to the emergency department after experiencing it. Two seizures following a severe headache. The patient’s history was negative for recent illnesses, head trauma, history of migraines, smoking, alcohol consumption, or intravenous drug use. The patient was diagnosed with CVST based on magnetic resonance venography (MRV). Genetic studies further identified homozygous mutations in the Prothrombin and MTHFR genes. Anticoagulant therapy was initiated with 60 mg of Enoxaparin twice daily and subsequently transitioned to Warfarin after 48 h continued for 3 months, and then replaced by rivaroxaban.

**Conclusions:**

This study highlights the importance of considering CVST as a cause of SAH, emphasizes the role of advanced imaging in diagnosis, and demonstrates a successful treatment approach using both traditional and direct oral anticoagulants. The insights provided in this article can contribute to improving the management of patients with CVST-related SAH.

## Introduction

Convexity subarachnoid hemorrhage (cSAH) is an uncommon presentation of subarachnoid hemorrhage, referring to bleeding more localized to the convexities of the brain, as opposed to spreading extensively throughout the subarachnoid space or involving interhemispheric fissures or basal cisterns [1, 2]. While ruptured cerebral aneurysms are common causes of SAH, cSAH typically results from bleeding from the superficial blood vessels on the brain's surface, and its characteristics may develop distinctly compared to typical SAH. It is associated with conditions such as cerebral amyloid angiopathy (CAA), venous thrombosis, or Dural arteriovenous fistulas [3]. Risk factors for convexity subarachnoid hemorrhage include advanced age, hypertension, and coagulation disorders [4].

Cerebral sinus thrombosis, also known as cerebral venous sinus thrombosis (CVST), is a condition characterized by the formation of blood clots within the dural venous sinuses of the brain [5, 6]. The diagnosis is usually made with imaging tests such as computed tomography (CT) scans or CT and magnetic resonance venography (MRV), which can identify the presence and location of bleeding in the subarachnoid space [7, 8].

Common signs of CVST include sudden and severe headaches, nausea, vomiting, seizures, confusion, and focal neurological deficits in the affected region [9]. Due to the specific location and causes of convexity subarachnoid hemorrhage, the management and prognosis may differ from other types of subarachnoid hemorrhage. It is essential for individuals who experience symptoms related to bleeding in the brain to seek immediate medical attention, as prompt diagnosis and treatment are crucial to prevent further complications and improve outcomes. Thus, the authors present a case and then discuss the pathology and management. The authors delineate a case study, subsequently elucidating the underlying pathophysiology and defining the management strategies.

## Patient Information

A 56-year-old female patient with a past medical history of hypertension and ischemic heart disease presented to the emergency department (ED) with a sudden onset of severe headache and two episodes of generalized tonic–clonic seizures. She was administered levetiracetam, an antiepileptic drug, and had no further seizures. She responded to verbal stimuli by opening her eyes, gave disoriented answers to the questions, and obeyed simple commands at arrival. Before loss of consciousness, the patient experienced a sudden, intense headache. Each seizure lasts under a minute, followed by a post-ictal phase before being brought to the ED. Notably, the patient's sister had a history of cerebrovascular accidents. The patient’s history was negative for recent illnesses, head trauma, migraines, smoking, alcohol consumption, or intravenous drug use. Her blood pressure elevated to 170/110, but other vital signs were stable. The patient was drowsy and mildly disoriented. Due to the postictal phase with monoplegia of her left upper limb and no signs of nuchal rigidity. She described the headache as "10 out of 10" in intensity.

**Fig. 1:**
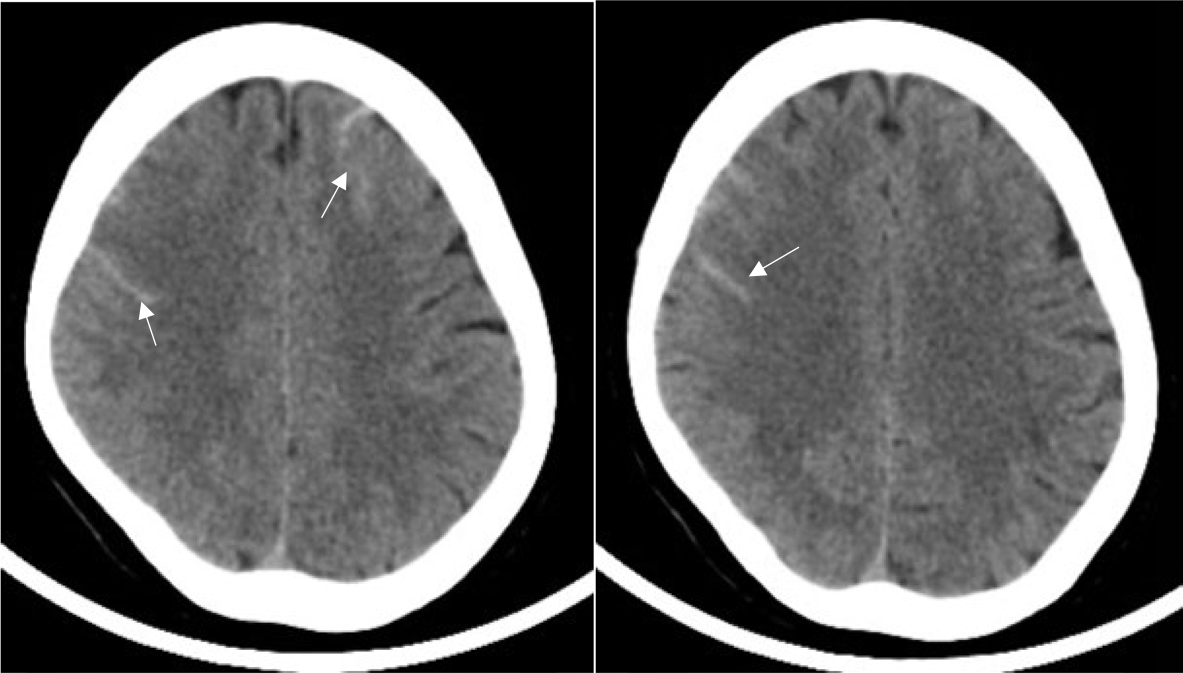
Brain CT scan in axial view. White arrows showing convexity subarachnoid hemorrhage

**Fig. 2:**
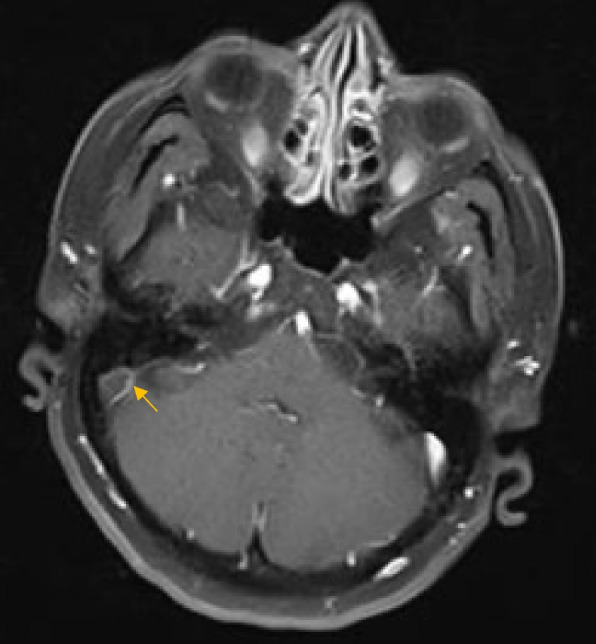
Contrast-enhanced T1-weighted MRI of the brain in axial plane showing thrombosis in the right transverse sinus

**Fig. 3:**
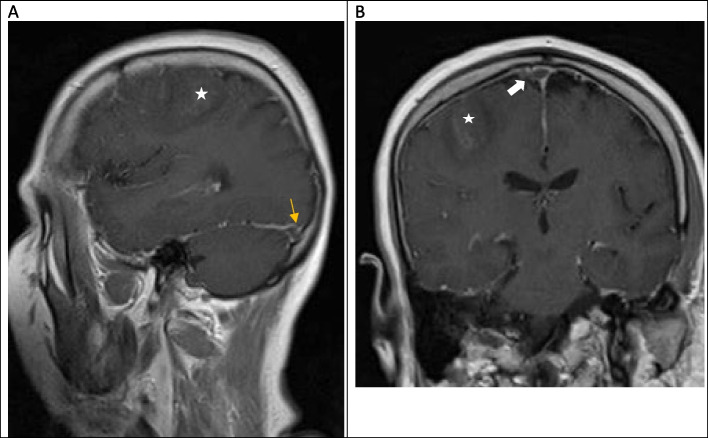
Contrast-enhanced T1-weighted MRI of the brain. **A** sagittal plane demonstrating a filling defect in the right transverse sinus. (Yellow arrow) and hypersignal area showing SAH (Red arrow) and surrounding hypodensity in favor of infarct (white star) **B** coronal plane (white arrow) showing thrombosis in the superior sagittal sinus

**Fig. 4:**
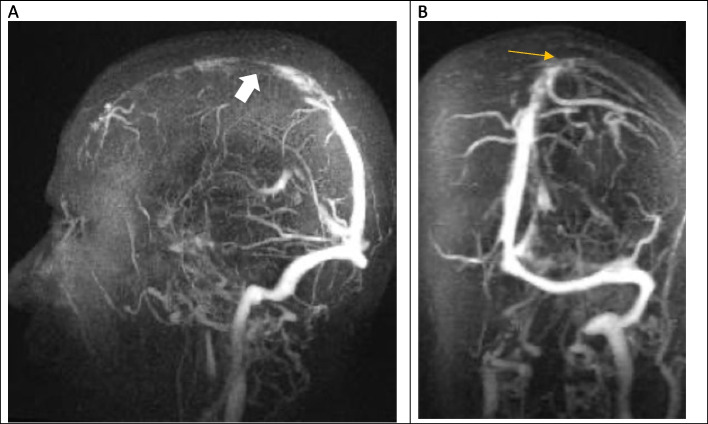
Contrast-enhanced MRV of the brain. **A** in the sagittal plane showing thrombosis in the superior sagittal sinus (white arrow), **B** in coronal plane showing thrombosis in the superior sagittal sinus and right transverse sinus. (Yellow arrow)

The CT scan showed convexity subarachnoid hemorrhage (Fig. 1). With clinical suspicion of CVST, the patient underwent MRI and MRV, which revealed thrombosis in the superior sagittal sinus and right transverse sinus (Figs. 2, 3 and 4). After performing a workup and obtaining an MRI scan, we diagnosed her with CVST. The initial MRA did not show evidence of cerebral vasoconstriction. We ruled out reversible cerebral vasoconstriction syndrome (RCVS) as a diagnosis and therefore did not perform digital subtraction angiography (DSA). Laboratory studies showed normal CBC, blood chemistry, and coagulation values, but a significant decrease in vitamin B12, low protein S, and increased C3 and C4 levels. Genetic studies revealed a homozygous mutation of Prothrombin (FII) 20210 G > A and Methylenetetrahydrofolate Reductase (MTHFR 677C > T, 1298 A > C).

**Fig. 5:**
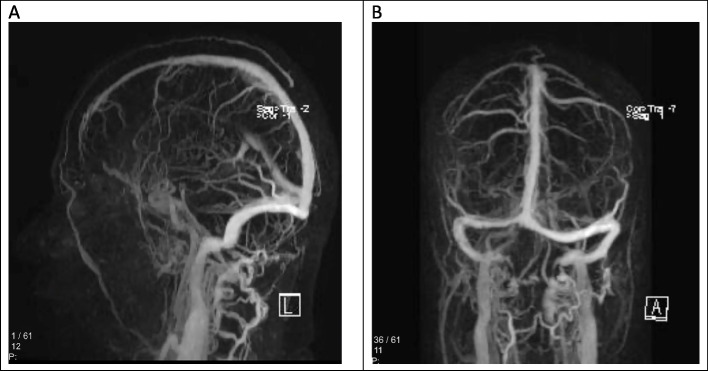
Contrast-enhanced MRV of the brain. **A** sagittal plane, **B** coronal plane showing Recanalization of thrombosis in the superior sagittal sinus and right transverse sinus

The patient was initially given 60 mg of Enoxaparin twice a day, then bridged to Warfarin after 48 h. After treatment initiation, the patient’s symptoms recovered gradually. She was discharged with an international normalized ratio (INR) of 2. After six months of treatment, we switched the patient from warfarin to rivaroxaban, a factor Xa inhibitor. This decision was based on the patient’s long-term anticoagulation requirement, the dietary limitations and frequent INR monitoring associated with warfarin, and the evidence of rivaroxaban’s efficacy and safety. After administration of rivaroxaban, follow-up imaging showed recanalization (Fig. 5), or restoration of blood flow, in the occluded vessel on the subsequent MRI examination. No aneurysm was detected in the follow-up imaging after six months.

## Discussion

In recent years, the diagnosis of CVST has increased, a trend attributed to heightened awareness and the availability of advanced non-invasive diagnostic tools such as MRI, which are instrumental in examining patients presenting with acute and subacute headaches and new-onset seizures [10]. Diagnosis is typically confirmed by detecting thrombosis in the cerebral veins and/or sinuses via venography or veno CT. Despite its generally favorable prognosis and the manifestation of a wide range of clinical symptoms, a significant number of CVST cases remain clinically unidentified [10, 11]. Historically considered life-threatening, the mortality rate associated with CVST has decreased by more than 50%, now ranging between 5–10% [12]. Predisposing factors are identifiable in up to 80% of patients [13]. with systemic diseases such as connective tissue disorders, other granulomatous or inflammatory conditions, and malignancies being the most common non-infectious causes. Headaches, a frequent complaint in emergency departments, are present in 70–90% of CVST cases, accounting for 2.6% of such visits [14, 15]. The MRV has largely supplanted invasive brain angiography and traditional CT scans, playing a pivotal role in the diagnosis and management of CVST [5]. The condition’s diverse clinical presentation makes CVST a challenging diagnosis, further complicated when associated with subarachnoid hemorrhage (SAH), which may manifest initially [16].

Subarachnoid hemorrhage (SAH) accounts for 5–10% of all strokes in the United States [13, 17]. Non-traumatic or spontaneous SAH is categorized into aneurysmal, non-aneurysmal, and peri-mesencephalic types, with intracranial aneurysm rupture being the predominant cause (80–85%), associated with high morbidity and mortality. Other etiologies include blood disorders, neoplasms, venous thrombosis, and unidentified aneurysms [17, 18]. Non-aneurysmal SAH typically follows a benign course with an excellent short-to-long-term prognosis [19]. The pathophysiology underlying non-aneurysmal SAH remains elusive, although research has indicated patterns of venous deviation in basal reservoirs leading to low-pressure bleeding [20]. The exact pathophysiological link between CVST and SAH is not fully understood. It is hypothesized that venous hemorrhagic infarction may lead to a secondary rupture in the subarachnoid space, causing SAH and that dural sinus thrombosis with subsequent venous hypertension may result in SAH due to the rupture of fragile cortical veins [16, 21]. Elevated plasma homocysteine levels have been reported as a potential connection between CVST and SAH, suggesting the need for further investigation into thrombophilia and pro-coagulative pathways to identify reversible causes such as hyperhomocysteinemia [22]. Cerebral Amyloid Angiopathy (CAA), characterized by amyloid beta-peptide deposits in the brain and leptomeningeal vessel walls, is a significant contributor to lobar intracerebral hemorrhage in older adults and accounts for 20% of SAH cases. Patients with risk factors such as advanced age, cognitive impairment, or Alzheimer’s disease should be evaluated for CAA-related SAH (cSAH) [23, 24].

The case series described patients experiencing severe headaches, seizures, and focal symptoms, including hemiparesis. The etiology of SAH in CVST may be linked to venous infarction and the rupture of delicate cortical veins. Localized SAH in CVST often occurs in proximity to the thrombosed venous structures. The administration of anticoagulant therapy in the treatment of cSAH within the context of CVST has yielded favorable outcomes for most patients. However, the mortality rate remains higher among individuals presenting with severe neurological deficits and complications such as parenchymal infarcts and elevated intracranial pressure [25]. A case series demonstrated the utilization of heparin followed by warfarin, with rivaroxaban employed in instances where INR control was challenging [26]. One relevant case report describes a 50-year-old male who presented with recurrent right focal clonic seizures and headaches. The key aspects of the diagnosis and treatment involved the use of advanced imaging (MRI with MRV) to confirm the underlying CVST, the management of seizures with antiseizure medications, and the initiation and titration of anticoagulation therapy to address the CVST and associated SAH [27]. Another case report discusses a 48-year-old woman presenting with SAH as the first manifestation of superior sagittal sinus thrombosis. The diagnosis was made using a combination of MRI and MRV, which revealed the presence of SAH and confirmed the underlying CVST. The patient was then managed with anticoagulation, which led to clinical improvement [28].

Khatib KI et al. have proposed a management algorithm for these patients, recommending that in the absence of mass effect or midline shift in neuroimaging, initial anticoagulant therapy should commence with heparin and low molecular weight heparin (LMWH) until the INR reaches therapeutic levels. Long-term oral anticoagulant (OAC) therapy is advised once the patient is capable of oral medication intake, and endovascular thrombectomy remains an option for cases contraindicated for anticoagulant use or when clinical status deteriorates rapidly due to elevated intracranial pressure (ICP) or venous thrombosis leading to venous infarcts [29]. Studies have indicated comparable safety and efficacy for rivaroxaban in contrast with standard anticoagulants [28, 29]. Nonetheless, there exists a paucity of clinical trials involving adult populations; hence, this article endeavors to bridge this gap. In the present case, rivaroxaban was selected due to the patient’s need for lifelong anticoagulation and poor adherence to dietary restrictions and warfarin monitoring, which exhibited no complications, including headaches, during the six-month follow-up period.

## Conclusion

In patients presenting with cSAH, it is crucial to consider CVST as a potential differential diagnosis. Neuroimaging and laboratory assessments, including genetic studies, should form the foundation for both the diagnosis and management of cSAH in the context of CVST. This study highlights the importance of considering CVST as a cause of SAH, emphasizes the role of advanced imaging techniques, such as MRI with MRV, in confirming the diagnosis, and demonstrates a successful treatment approach using both traditional anticoagulants (enoxaparin and warfarin) and direct oral anticoagulants (rivaroxaban). The insights provided in this article can contribute to improving the management of patients with CVST-related SAH.

## Data Availability

The datasets used and/or analyzed during the current study are available from the corresponding author upon reasonable request.

## Abbreviations

SAH: Subarachnoid hemorrhage
cSAH: Convexity subarachnoid hemorrhage
CAA: Cerebral amyloid angiopathy
CVST: Cerebral venous sinus thrombosis
CT: Computed tomography
MRI: Magnetic resonance imaging
MRA: Magnetic resonance angiography
MRV: Magnetic resonance venography
RCVS: Reversible cerebral vasoconstriction syndrome
DSA: Digital subtraction angiography
ED: Emergency department
MTHFR: Methylenetetrahydrofolate Reductase
INR: International normalized ratio
LMWH: Low molecular weight heparin
OAC: Oral anticoagulant
cSAH: CAA-related SAH

## Data availability

No datasets were generated or analysed during the current study.
